# The relationships between faecal egg counts and gut microbial composition in UK Thoroughbreds infected by cyathostomins

**DOI:** 10.1016/j.ijpara.2017.11.003

**Published:** 2018-05

**Authors:** L.E. Peachey, R.A. Molena, T.P. Jenkins, A. Di Cesare, D. Traversa, J.E. Hodgkinson, C. Cantacessi

**Affiliations:** a Department of Veterinary Medicine, University of Cambridge, Madingley Road, CB3 0ES, United Kingdom; b Faculty of Veterinary Medicine, University of Teramo, Teramo, 64100, Italy; c Department of Infection Biology, University of Liverpool, Leahurst, Neston CH64 7TE, United Kingdom

**Keywords:** Helminth-microbiota interactions, Thoroughbred horses, Cyathostomins, 16S rRNA sequencing, *Adlercreutzia*, Methanomicrobia, TM7

## Abstract

A growing body of evidence, particularly in humans and rodents, supports the existence of a complex network of interactions occurring between gastrointestinal (GI) helminth parasites and the gut commensal bacteria, with substantial effects on both host immunity and metabolic potential. However, little is known of the fundamental biology of such interactions in other animal species; nonetheless, given the considerable economic losses associated with GI parasites, particularly in livestock and equines, as well as the global threat of emerging anthelmintic resistance, further explorations of the complexities of host-helminth-microbiota interactions in these species are needed. This study characterises the composition of the equine gut commensal flora associated with the presence, in faecal samples, of low (C*low*) and high (C*high*) numbers of eggs of an important group of GI parasites (i.e. the cyathostomins), prior to and following anthelmintic treatment. High-throughput sequencing of bacterial 16S rRNA amplicons and associated bioinformatics and statistical analyses of sequence data revealed strong clustering according to faecal egg counts (*P* = 0.003). A trend towards increased populations of Methanomicrobia (class) and *Dehalobacterium* (genus) was observed in C*low* in comparison with C*high*. Anthelmintic treatment in C*high* was associated with a significant reduction of the bacterial Phylum TM7 14 days post-ivermectin administration, as well as a transient expansion of *Adlercreutzia* spp. at 2 days post-treatment. This study provides a first known insight into the discovery of the intimate mechanisms governing host-parasite-microbiota interactions in equines, and sets a basis for the development of novel, biology-based intervention strategies against equine GI helminths based on the manipulation of the commensal gut flora.

## Highlights

• We profiled the faecal microbial communities of horses with cyathostomin infections, pre- and post-anthelmintic treatment.
• Methanomicrobia and *Dehalobacterium* were expanded in the microbiota of horses with low cyathostomin faecal egg counts.
• A reduction in TM7 and an expansion in *Adlercreutzia* followed anthelmintic treatment in horses with high faecal egg counts.
• Novel intervention strategies against cyathostomins based on the manipulation of the gut flora may be developed.

## Graphical abstract

**Figure:**
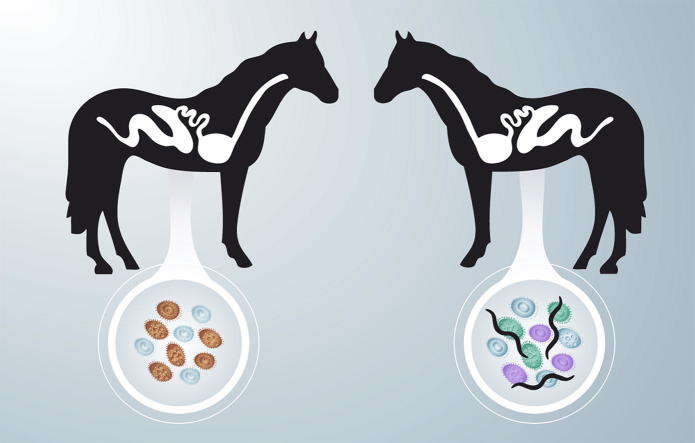

## 1 Introduction

Cyathostomins are amongst the most important intestinal nematodes of horses globally (Love et al., 1999, Matthews, 2011, Stratford et al., 2011) with reported prevalence rates as high as 89–100% in equine herds (Mfitilodze and Hutchinson, 1990, Collobert-Laugier et al., 2002, Hinney et al., 2011, Morariu et al., 2016). Clinical signs of cyathostomin infection range from non-specific weight loss to colic and colitis caused by mass emergence of larvae from the large intestinal wall (= larval cyathostominosis), which may prove fatal (Uhlinger, 1991, Murphy and Love, 1997, Lyons et al., 2000, Peregrine et al., 2006). Young Thoroughbred (TB) stock kept in herds are at high risk of developing serious complications of infection, and hence the implementation of effective strategies of parasite control is a top priority for the TB industry. Control of cyathostomin infections has traditionally relied on the regular administration of chemotherapeutic drugs (i.e. anthelmintics); however, the frequent and uncontrolled use of these compounds has led to the global emergence of resistant populations of parasites (Nielsen et al., 2014, Peregrine et al., 2014). In particular, foci of multi-drug resistance have been recently reported in TB stud farms in the United Kingdom (Relf et al., 2014). This observation, coupled with the lack of novel anthelmintic compounds licenced for use in equids, represents a ‘Damocle’s sword’ for the UK (and global) equine industry. Therefore, alternative strategies for parasite control are urgently needed; in order to support the discovery of such strategies, a deeper understanding of the complex interactions occurring at the host-parasite interface, particularly at the site/s of infection (i.e. the gut), is required.

While a multitude of factors is responsible for the host-parasite interactions which determine infection outcome, increasing attention is being paid to the complex interplay between gastrointestinal (GI) parasites and the host commensal gut flora (Bancroft et al., 2012, Glendinning et al., 2014). Indeed, recent studies have reported significant fluctuations in the composition of the vertebrate gut microbiota associated with helminth infections, that were accompanied by shifts in both systemic and local immunity (Bancroft et al., 2012, Leung and Loke, 2013, Fricke et al., 2015, Houlden et al., 2015, Cattadori et al., 2016, Gause and Maizels, 2016). However, thus far, knowledge of helminth–microbiota cross-talk relies heavily on studies conducted in humans and/or rodent models of infection and disease (Walk et al., 2010, Rausch et al., 2013, Cantacessi et al., 2014, Lee et al., 2014, Reynolds et al., 2014a, Fricke et al., 2015, Giacomin et al., 2015, Giacomin et al., 2016, Holm et al., 2015, Houlden et al., 2015, Kreisinger et al., 2015, McKenney et al., 2015, Cattadori et al., 2016). In particular, while only a handful of studies to date have characterised the composition of the gut microbiota of veterinary species infected by GI helminths (Li et al., 2011, Li et al., 2012, Li et al., 2016, Wu et al., 2012, Slapeta et al., 2015, Duarte et al., 2016; reviewed by Peachey et al., 2017), no data is currently available on the effects of infections by GI helminths such as cyathostomins on the composition of the equine commensal flora. Acquiring this fundamental knowledge will be key to the development of novel holistic approaches to equid parasite control aimed at improving host reponses to infections. In this study, we characterise the gut microbial profiles of a cohort of UK TB broodmares with low and high numbers of cyathostomin eggs in faeces (as determined by faecal egg count (FEC) analysis), and examine the effects that administration of a commonly used anthelmintic, i.e. ivermectin, exerts on the overall composition of the gut microbiota as well as relative abundances of individual microbial species.

## 2 Materials and methods

### 2.1 Ethics statement

This study was approved and carried out in strict accordance and compliance with the guidelines of the Institutional Ethical Review Committee, Department of Veterinary Medicine, University of Cambridge, UK (Research Project No. CR190). Written informed consent was obtained from the stud farm from which study samples were collected.

### 2.2 Sampling and diagnostic procedures

For this study, a cohort of TB broodmares was recruited from a stud farm in eastern England, UK. The stud hosts approximately 130 pregnant broodmares each year, which are kept at pasture in groups of 2–8 across 480 hectares. All broodmares are subjected to targeted anthelmintic treatments (ivermectin and moxidectin), based on FEC measurements at 3 monthly intervals. In addition, praziquantel is administered to each broodmare three times a year for tapeworm control, whilst a single moxidectin treatment is administered in late November for control of encysted cyathostomin larvae. Samples used in this study were collected in September-October 2016; all broodmares had received ivermectin and praziquantel in May and August 2016, respectively. A total of 117 TB pregnant broodmares, between 5 and 8 months of gestation at the time of sampling, were screened for infection by cyathostomins. Briefly, individual faecal samples were collected on three consecutive days over a 7 day period; aliquots of each sample were subjected to (i) FEC analysis using a centrifugal floatation technique sensitive to one egg per gram (e.p.g.) (Christie and Jackson, 1982), and (ii) screening for infections with the common equine cestode *Anoplocephala perfoliata* using a double sugar flotation technique (Rehbein et al., 2011). Upon observation of strongyle eggs during FEC analysis, the remaining faecal aliquots were subjected to larval culture to allow for subsequent identification of infecting nematode species using an established Reverse Line Blot (RLB) hybridisation method (Traversa et al., 2007, Cwiklinski et al., 2012). Briefly, genomic DNA was extracted from individual L3s harvested from each larval culture, and the intergenic spacer (IGS) region was amplified by nested PCR using conserved biotin labelled primers (Traversa et al., 2007). The PCR products were then incubated with biodyne C membrane-bound specific DNA probes for 21 different cyathostomin species (Cwiklinski et al., 2012), incubated with extravidin peroxidase and visualised using x-ray film. Horses were recruited in our study if they satisfied the following criteria: (i) FEC of ≥100 e.p.g. (= C*high*) or ≤10 e.p.g. (C*low*) in three consecutive samples collected over a 7 day period; (ii) matched by approximate age and paddock; (iii) negative for co-infections with other GI helminths; (iv) no antibiotic treatment for at least 2 months prior to sampling; and (v) no previous anthelmintic treatment other than praziquantel for at least 4 months prior to sampling. Horses enrolled were kept at pasture for the duration of the study and fed 1 kg of custom concentrate mix daily.

### 2.3 Anthelmintic treatment

Individual, naturally voided, faecal samples were collected from the centre of the faecal mass from C*high* and C*low* animals, as well as from three non-pregnant broodmares on day 0 (D0). Then, an anthelmintic treatment (Eqvalan: ivermectin 0.2 mg/kg) was immediately administered to each animal. Sampling was repeated as above at day 2 (D2) and day 14 (D14) post-treatment (p.t.). A 100 g aliquot of each faecal sample was snap frozen, transported to the laboratory and stored at −20 °C within 2 h of collection, prior to genomic DNA extraction and high-throughput sequencing of a hypervariable region of the bacterial 16S rRNA gene (see Section 2.4), while the remainder was kept fresh and subjected to FEC analysis as described above.

### 2.4 High-throughput 16S rRNA sequencing

Genomic DNA was extracted from individual faecal samples, as well as from five negative ‘blank’ (= no DNA) controls, using the PowerSoil® DNA Isolation Kit (Qiagen, Carlsbad, CA, USA), according to the manufacturers’ instructions. Microbial communities in each sample were identified *via* Illumina high-throughput sequencing of the V3-V4 hypervariable region of the bacterial 16S rRNA gene. In particular, the V3-V4 region was PCR-amplified using universal primers (Forward, 5′-TCG TCG GCA GCG TCA GAT GTG TAT AAG AGA CAG CCT ACG GGN GGC WGC AG-3′; Reverse, 5′-GTC TCG TGG GCT CGG AGA TGT GTA TAA GAG ACA GGA CTA CHV GGG TAT CTA ATC C-3′) (Klindworth et al., 2013), that contained Illumina (San Diego, California, USA) adapter over-hang nucleotide sequences, using the NEBNext® Q5® Hot Start HiFi DNA polymerase (New England Biolabs® Inc, Massachusetts, USA). For PCR amplification, the following thermocycling protocol was used: 98 °C/2 min, 20 cycles of 98 °C/15 s, 63 °C/30 s, and 72 °C/30 s, and 72 °C/5 min. Amplicons were purified using AMPure XP PCR Purification beads (Beckman Coulter, Brea, California, USA). The index PCR was performed using the NEBNext hot start high-fidelity DNA polymerase and Nextera XT index primers (Illumina) according to the following thermocycling protocol: 98 °C/30 s, 8 cycles of 98 °C/10 s, 65 °C/75 s and at 65 °C/5 min. The indexed samples were purified using AMPure XP beads and quantified using the Qubit Quant-iTTM dsDNA Broad-Range Assay Kit (Life Technologies, Carlsbad, California, USA). Then, equal quantities from each sample were pooled and the resulting library was quantified using the NEBNext® Library Quant Kit for Illumina® (New England Biolabs® Inc). High-throughput sequencing was performed on an Illumina MiSeq platform using the v3 chemistry (301 bp paired-end reads).

### 2.5 Bioinformatics analyses

Following trimming of primer sequences using Cutadapt (https://cutadapt.readthedocs.org/en/stable/), raw paired-end Illumina reads were joined using the Quantitative Insights Into Microbial Ecology (QIIME) software suite (version 1.9.0) (Caporaso et al., 2010) and quality filtered using the ‘usearch_qf’ script with default settings. Then, high-quality sequences were clustered into Operational Taxonomic Units (OTUs) on the basis of similarity to bacterial sequences available in the Greengenes database (v13.8; http://greengenes.secondgenome.com/; 97% sequence similarity cut-off) using the UCLUST software; sequences that could not be matched to references in the Greengenes database were clustered de novo based on pair-wise sequence identity (97% sequence identity cut-off) (cf. Duarte et al., 2016). Singleton OTUs and OTUs assigned to sequences obtained from no-DNA control samples were subtracted from individual datasets prior to downstream analysis. For normalisation, a subsampled OTU table was generated by random sampling (without replacement) of the input OTU table using an implementation of the Mersenne twister algorithm (http://www.numpy.org). Cumulative-sum scaling (CSS) and log2 transformation were applied to account for the non-normal distribution of taxonomic counts data. Statistical analyses were conducted on the Calypso platform (cgenome.net/calypso/); samples were clustered using supervised Canonical Correspondence Analysis (CCA) including FEC (C*high*:C*low*) and time-point (D0, D2 and D14 p.t.) as explanatory variables. Differences in bacterial alpha diversity (Shannon diversity) between groups were evaluated using a paired t-test or ANOVA (depending on the number of groups for comparison). Beta diversity of microbial communities was calculated using weighted UniFrac distances and, based on the matrices, differences in beta diversity between groups were calculated using Permutational Analysis of Multivariate Dispersions (PERMDISP) through the ‘betadisper’ function (Anderson et al., 2006). Differences in the relative abundances of individual microbial species between groups were assessed using the Linear discriminant analysis Effect Size (LEfSe) workflow (Segata et al., 2011), by assigning FEC/timepoint ‘groupings’ as the comparison class. All statistical analyses were repeated on a sub-group of horses with FEC ≥200 e.p.g. (*n* = 8) and 0 e.p.g. (*n* = 7), hereafter referred to as ‘C*200*’ and ‘C*0*’, respectively. *P* < 0.05 was considered statistically significant.

## 3 Results

### 3.1 Prevalence of cyathostomin infection and group selection

Of 117 broodmares examined for cyathostomin infection, 36 matched the criteria outlined in Section 2.2*.* and were therefore enrolled in this study. Of these, 18 broodmares had an average FEC of ≥100 e.p.g. (range 100–418), and were thus enrolled as C*high* (Table 1). Amongst these, eight were defined as C*200* based on average FEC of ≥200 e.p.g. (Table 1). Conversely, 18 broodmares with FEC of ≤10 e.p.g. (range 0–10) were enrolled as C*low* and, of these, seven could be included as C*0* (Table 1). While no known effect size estimates for changes in microbiota due to parasitism in horses are currently available, data from previous studies in other host-helminth systems (effect size: 1.5; cf. Giacomin et al., 2015, 2016) provided us with 84% power to detect changes in gut microbial composition between groups (*n* = 18 in each C*high* and C*low* group) using canonical analysis of principal coordinates.

**Table 1:** Faecal egg counts (FEC) recorded from C*high* (FEC ≥100 eggs per gram, e.p.g.) and C*low* (FEC ≤10 e.p.g.) broodmares enrolled in the study, as well as from non-pregnant controls (NPC), over three consecutive samplings performed pre-anthelmintic treatment (Day 0 (D0)), as well as at 2 and 14 days post-treatment (D2, D14). Horses with FEC ≥200 e.p.g. (C*200*) and 0 e.p.g. (C*0*), are indicated in bold.

Group	Animal I.D.	Age (years)	Tapeworm FEC (e.p.g.)	Ascarid FEC (e.p.g.)	Mean consecutive strongyle FEC (e.p.g.) (±S.E.) D0	FEC D2 (e.p.g.)	FEC D14 (e.p.g.)
C*high*							
	MA	6	0	0	123 (±19)	0	0
	CT	5	0	0	130 (±14)	0	0
	LE	10	0	0	113 (±17)	0	0
	PB	5	0	0	171 (±15)	0	0
	SC	4	0	0	101 (±8)	2 (±2)	0
	MSJ	6	0	0	100 (±6)	0	0
	WD	7	0	0	128 (±16)	0	0
	NS	8	0	0	120 (±11)	1 (±1)	0
	HY	7	0	0	150 (±14)	0	0
	RM	4	0	0	139 (±49)	0	0
	**HS**	**6**	**0**	**0**	**200 (±39)**	**1 (±1)**	**1 (±1)**
	**HT**	**6**	**0**	**0**	**228 (±22)**	**0**	**0**
	**NO**	**6**	**0**	**0**	**271 (±17)**	**0**	**1 (±1)**
	**QM**	**4**	**0**	**0**	**418 (±112)**	**0**	**0**
	**SB**	**8**	**0**	**0**	**206 (±37)**	**1 (±1)**	**1 (±1)**
	**TC**	**4**	**0**	**0**	**235 (±12)**	**0**	**1 (±1)**
	**MQ**	**6**	**0**	**0**	**228 (±22)**	**0**	**0**
	**VR**	**4**	**0**	**0**	**279 (±61)**	**1 (±1)**	**0**
C*low*							
	GL	4	0	0	10 (±2)	0	0
	DWD	8	0	0	0.3 (± 0.3)	0	0
	LVN	4	0	0	5 (±1)	0	0
	BB	7	0	0	1 (±1)	0	4 (±1)
	LF	5	0	0	10 (±2)	0	0
	IR	7	0	0	3 (±1)	0	0
	DDR	8	0	0	1 (±1)	0	0
	LAL	5	0	0	0.3 (±0.3)	0	0
	PT	7	0	0	0.3 (±0.3)	0	0
	PP	6	0	0	2 (±1)	0	0
	MG	5	0	0	0.5 (±0.3)	0	0
	**SY**	**8**	**0**	**0**	**0**	**0**	**0**
	**BX**	**16**	**0**	**0**	**0**	**0**	**0**
	**MR**	**10**	**0**	**0**	**0**	**0**	**0**
	**DD**		**0**	**0**	**0**	**0**	**0**
	**ED**	**8**	**0**	**0**	**0**	**0**	**0**
	**EP**	**5**	**0**	**0**	**0**	**0**	**0**
	**SWC**	**8**	**0**	**0**	**0**	**0**	**0**
*NPC*							
	BET	8	0	0	412 (±45)	-	-
	BL	12	0	0	22 (±1)	-	-
	ST	10	0	0	0	-	-

Larval culture followed by species identification via RLB revealed infections with the following cyathostomin species: *Cyathostomum (Cya.) catinatum* (88%), *Cylicostephanus (Cys.) longibursatus* (71%), *Cylicocyclus (Cyc.) nassatus* (59%), *Coronocyclus coronatus* (41%), *Cylicocyclus calicatus* (41%), *Cylicocyclus radiatus* (29%), *Cylicostephanus goldi* (24%), *Cylicostephanus leptostomus* (18%), *Cyathostomum pateratum* (18%), *Cylicocyclus ashworthi* (18%) and *Cylicocyclus insigne* (12%). FEC analysis performed on samples collected at D2 and D14 p.t. showed FEC reduction rates (FECR) of >95% in all treated animals (Table 1).

### 3.2 Microbiota profiling

**Fig. 1:**
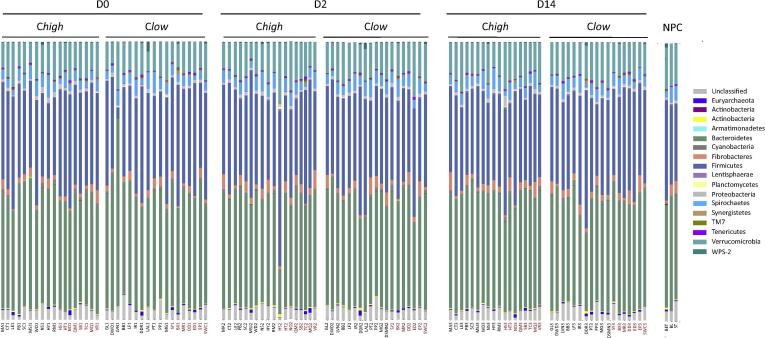
Bar charts depicting the relative abundances of faecal bacterial phyla from broodmares with faecal egg counts (FEC) ≥100 eggs per gram (e.p.g.) (= C*high*) and ≤10 e.p.g. (= C*low*), according to sampling time point (i.e. pre-anthelmintic treatment (Day 0 (D0), and 2 and 14 days post-treatment (D2 and D14, respectively)), and from non-pregnant controls (NPC). Samples from broodmares with FEC ≥200 e.p.g. (*C200*) and 0 (*C0*) are indicated in red, while remaining samples are indicated in black.

A total of 39,461,550 raw paired-end reads were generated from DNA faecal extracts of C*high* and C*low* broodmares and subjected to further processing. Following primer trimming, joining of paired-end reads, filtering of low-quality sequences and removal of ‘contaminant’ and singleton OTUs, a total of 8,077,490 high quality sequences (mean = 73,423 ± 3,610) were retained for further bioinformatics analysis (not shown). Raw and curated sequence data generated in this study are available from Mendeley Data at http://doi.org/10.17632/g7chkjrp8f.1. The rarefaction curves generated following in-silico subtraction of low-quality and contaminant sequences indicated that the vast majority of faecal bacterial communities were represented in the remaining sequence data, thus allowing us to undertake further analyses. These sequences were assigned to 95,286 OTUs and 15 bacterial phyla, respectively. The Phyla Bacteroidetes (39.9%) and Firmicutes (34.0%) were predominant in all samples, followed by the Phyla Verrucomicrobia (12.0%), Spirochaetes (3.9%), Fibrobacteres (2.4%), Cyanobacteria (1%), Proteobacteria (0.9%), Euryarcheota (0.4%), Tenericutes (0.4%), TM7 (0.3%), Actinobacteria (0.3%), Lentisphaerae (0.3%), Synergistetes (0.2%), WPS-2 (0.2%) and Planctomycetes (0.1%) (Fig. 1), while 3.4% of OTUs could not be assigned to any bacterial group. Predominant sub-taxa were Bacteroidia (class), Bacteroidales (order) and Bacteroidales (family) within the Phylum Bacteroidetes, and Clostridia (class), Clostridiales (order) and Ruminococcae (family) within the Firmicutes (Fig. 1). Two samples, LVN1 and HS2, differed markedly in the relative proportions of the two most abundant phyla, Bacteroidetes and Firmicutes, when compared with samples from other broodmares (Fig. 1), likely indicating dysbiosis. Therefore, in order to reduce biases due to these potential ‘outliers’, these samples were excluded from further statistical analyses (Fig. 1).

### 3.3 Differences in microbial composition between C*high* and C*low*, and pre- and post-anthelmintic treatment

**Fig. 2:**
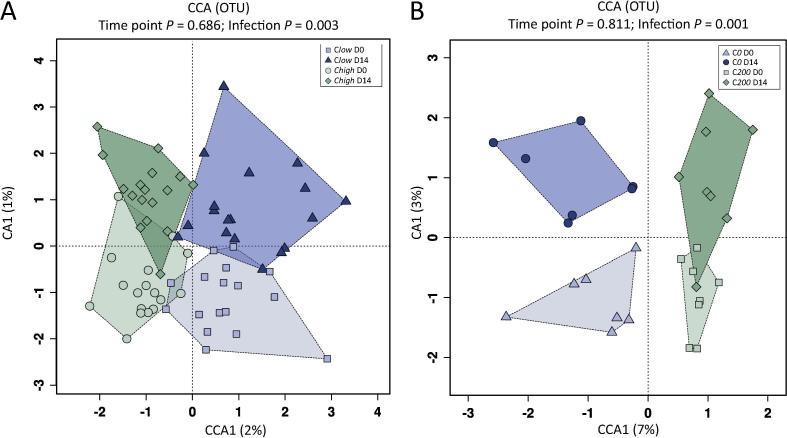
The microbial composition of faecal samples ordered by supervised Canonical Correspondence Analysis (CCA) from broodmares with (A) faecal egg counts (FEC) ≥100 eggs per gram (e.p.g.) (= C*high*) and ≤10 e.p.g. (= C*low*), pre-anthelmintic treatment (Day 0 (D0)) and at 14 days post-treatment (D14) (B) with FEC ≥200 e.p.g. (C*200*) and 0 (C*0*) at D0 and D14. OTU, Operational Taxonomic Unit.

Microbial community profiles of each sample were grouped by hierarchical clustering and ordinated by supervised CCA. Using these methods, a significant association was observed between microbial composition and FEC (C*high* versus C*low*) (*P* = 0.003), while clustering according to time point pre- and post- anthelmintic treatment (D0 versus D14) did not reach statistical significance (*P* = 0.686) (Fig. 2A). CCA of C*200* versus C*0* led to a clear separation according to FEC (*P* = 0.001), whilst the effect of anthelmintic treatment remained insignificant (*P* = 0.811) (Fig. 2B).

**Fig. 3:**
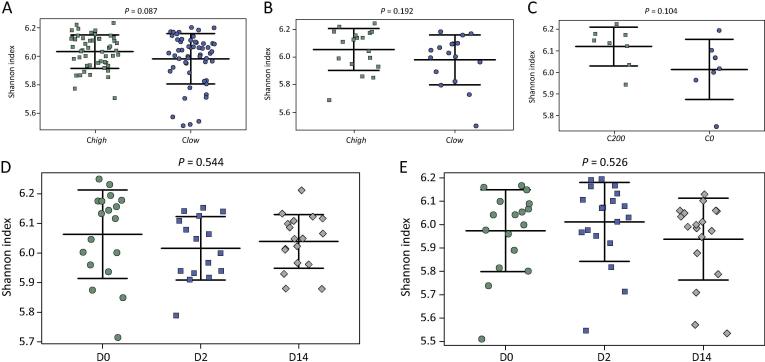
Shannon diversity charts comparing faecal microbial alpha diversity of broodmares (A) with faecal egg counts (FEC) ≥100 eggs per gram (e.p.g.) (= C*high*) and ≤10 e.p.g. (= C*low*) at all time points (i.e. pre-anthelmintic treatment (D0) and 2 and 14 days post-treatment (D2 and D14, respectively)), (B) C*high* and C*low* at D0 only, (C) with FEC ≥200 e.p.g. (C*200*) and 0 (C*0*) at D0 only, and (D) C*high* and (E) C*low* at D0, D2 and D14.

**Fig. 4:**
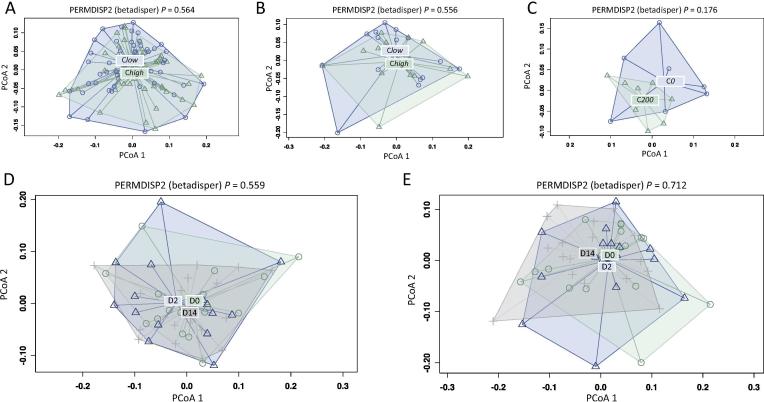
Permutational Analysis of Multivariate Dispersions (PERMDISP) plots comparing the faecal microbial beta diversity of broodmares (A) with faecal egg counts (FEC) ≥100 eggs per gram (e.p.g.) (C*high*) and ≤10 e.p.g. (C*low*) at all time points (i.e. pre-anthelmintic treatment (D0) and 2 and 14 days post-treatment (D2 and D14, respectively)), (B) C*high* and C*low* at D0 only, (C) with FEC ≥200 e.p.g. (C*200*) and 0 (C*0*) at D0 only, and (D) C*high* and (e) *Clow* at D0, D2 and D14. PCoA, principal coordinate analysis.

No significant differences in OTU alpha diversity (Shannon Index) were recorded between C*high* and C*low*, or between samples collected at D0, D2 and D14 p.t. (Fig. 3A–C). A trend towards increased alpha diversity in C*high* versus C*low* at all time-points was observed (*P* = 0.087) (Fig. 3A). This trend was also observed when C*200* samples were compared with C*0* at D0, despite smaller group sizes (*P* = 0.102) (Fig. 3C). No significant differences in beta diversity, as measured by PERMDISP, were observed between groups (Fig. 4).

**Fig. 5:**
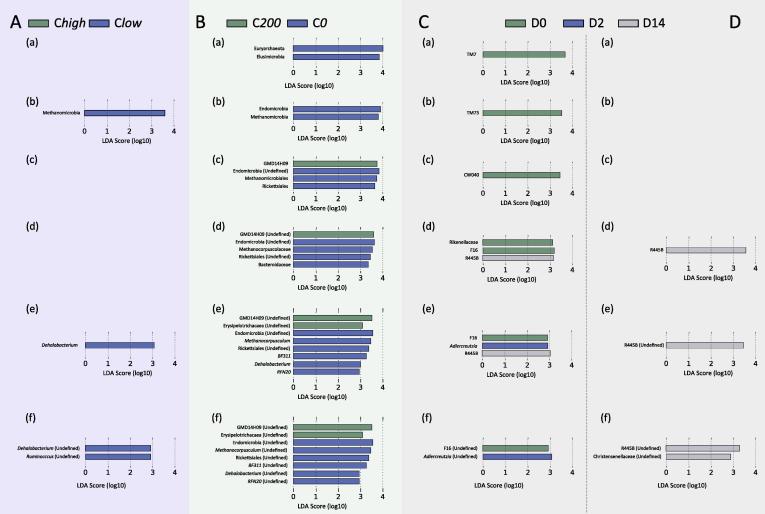
Linear discriminant analysis effect size bar charts depicting differences in abundance of individual bacterial taxa at the phylum, class, order, family, genus and species levels in faecal samples from broodmares (A) with faecal egg counts (FEC) ≥100 eggs per gram (e.p.g.) (C*high*) and ≤10 e.p.g. (C*low*), (B) with FEC ≥200 e.p.g. (*C200*) and 0 (*C0*) at Day 0 (D0), and in (C) *Chigh* and (D) C*low* according to sampling time point (i.e. pre-anthelmintic treatment (D0) and 2 and 14 days post-treatment (D2 and D14, respectively)). LDA, linear discriminant analysis.

Differences in abundance of individual taxa at the phylum, class, order, family, genus and species level were detected between C*high* and C*low* samples, as well as between samples collected at D0, and D2 and D14 p.t. (Fig. 5). Samples from C*low* at D0 (pre-treatment) were characterised by an increased abundance of Methanobacteria (class), *Dehalobacterium* (genus) and unclassified *Dehalobacterium* and *Ruminococcus* (species) compared with samples from C*high* (Fig. 5A). The same taxa were increased in C*0* compared with C*200*, with the addition of methanogens of the Family Methanocorpusculacaea belonging to Order Methanomicrobiales, Class Methanobacteria, Phylum Euryarchaeota; Order Endomicrobiales, Phylum Elusimicrobia; Rickettsiales (order, family, genus, species); Family Bacteroidaceae, genus BF311 and species RFN20 (Fig. 5B). The taxa GMD14H09 (order, family, genus, species) of the Phylum Proteobacteria were increased in samples from C*200* compared with C*0* (Fig. 5B). Anthelmintic treatment in C*high* was accompanied by a decrease in the Phylum TM7 at D14, when compared with pre-treatment samples (Fig. 5C). Additionally, the taxa *Adlercreutzia* and R445B were increased at D2 and D14, respectively, compared with D0 samples (Fig. 5C). In C*low*, treatment was also associated with an increase in R445B (family, genus, species) at D14 (Fig. 5D).

## 4 Discussion

This study is the first known to report a significant association between numbers of cyathostomin eggs in faecal samples from UK Thoroughbreds and the composition of the host gut microbiota. A particularly significant shift in microbial profiles was observed when the faecal bacterial populations of a group of broodmares with FEC of ≥200 e.p.g. were compared with those with observed FEC of 0. These data are consistent with observations from published studies in both humans and other veterinary species, including rodent models of infection and disease (Lee et al., 2014, Holm et al., 2015, Houlden et al., 2015, McKenney et al., 2015, Duarte et al., 2016, Li et al., 2016). In addition, the administration of a routinely used anthelmintic (i.e. ivermectin) to both C*high* and C*low* resulted in further progressive changes of the microbial profiling of treated horses. While such changes did not reach statistical significance when analysed using a multivariate model, this trend suggests that parasite-associated modifications in the composition of the host gut microbiota may be transient, and dependent on the presence of live infections, a hypothesis which requires thorough testing.

Overall, the bacterial phyla identified in this study were consistent between groups of animals enrolled; this observation differs from the results of previous studies that had reported significant variability in faecal microbial profiling between horses, largely related to variations in diet and age, and the presence of underlying diseases (Costa et al., 2012, Daly et al., 2012, Steelman et al., 2012, O’Donnell et al., 2013, Dougal et al., 2014, Fernandes et al., 2014, Weese et al., 2014). Thus, our finding likely indicates that the impact of such confounding factors was successfully minimised by our study design, and that the recorded differences in microbial composition were indeed associated with parasite infections. Bacteria belonging to the Phylum Bacteroidetes were predominant in animals examined in our study; conversely, other investigations had reported Firmicutes as being the most prevalent phylum in the horse gut flora (Costa et al., 2012, Costa et al., 2015a, Costa et al., 2015b, Shepherd et al., 2012, Dougal et al., 2014, Fernandes et al., 2014, Weese et al., 2014, Proudman et al., 2015). Dietary differences between horse cohorts enrolled in this and previous studies are likely to be responsible for this discrepancy (cf. Daly et al., 2012, Fernandes et al., 2014).

Overall, a trend towards increased microbial alpha diversity, i.e. the number of different OTUs in each sample (‘richness’) and their relative abundance (‘evenness’) (Tuomisto, 2010), was observed in samples from C*high* compared with those from C*low* at D0 (pre-anthelmintic treatment) and in C*200* versus C*0*, although these differences did not reach statistical significance. Nevertheless, this observation is supported by the results of a number of previous studies in other host-helminth systems, in which the establishment of parasitic infections was associated with an overall increase in alpha diversity of the gut microbiota (Broadhurst et al., 2012, Lee et al., 2014, Giacomin et al., 2015, 2016). Given that a number of inflammatory GI and/or systemic diseases are accompanied by a reduced alpha diversity (Manichanh et al., 2006, Sepehri et al., 2007, Abrahamsson et al., 2012, Abrahamsson et al., 2014), the increase in GI microbial diversity observed in the presence of helminth infections has been hypothesized to represent one of the possible mechanisms by which parasites suppress host inflammatory responses, thus ensuring their long-term survival in the host gut (Bancroft et al., 2012, Glendinning et al., 2014). Therefore, the trends towards increased alpha diversity observed in the faecal microbiota of horses moderately infected by cyathostomins may also result from an increase in gut homeostasis promoted by the parasites. Future studies evaluating the prevalence and incidence of equine inflammatory diseases (e.g. inflammatory bowel disease and recurrent airway obstruction) in the presence or absence of parasitic infections could represent significant first steps in this area of research.

In addition to global microbial diversity, significant variations in the abundance of specific bacterial taxa were observed between groups. In particular, a higher abundance of microorganisms belonging to the Class Methanomicrobia was observed in C*low* (D0) when compared with C*high*. This difference was exacerbated in C*200* versus C*0*, with further significant increases in methanogens belonging to Class Methanomicrobia recorded in C*0*, thus suggesting a negative correlation between methanogen abundance and FEC. Methanomicrobia belong to the Phylum Euryarchaeota, Kingdom Archaea and are phylogenetically distinct from bacteria and eukaryotes, although they retain the prokaryote 16S rRNA gene (Woese and Gupta, 1981, Winker and Woese, 1991). Particularly in ruminants, the role of the Archaeal methanogens in the digestion of fibre has been well documented (St-Pierre et al., 2015). In equids, little is known of the functional diversity of methanogens; however, consistent with our findings, a recent study reported Methanomicrobiales as being predominant in the horse gut (Lwin and Matsui, 2014). The underlying mechanisms by which GI helminths may be promoting a reduction in populations of methanogens are unclear. Similarly to hypotheses formulated for other host-helminth systems, cyathostomins may prevent expansion of methanogens directly, e.g. via their excretory-secretory products, or indirectly via parasite-induced changes in mucosal immunity (reviewed by Peachey et al., 2017). Alternatively, a high abundance of methanogens prior to helminth infections may bias host immune responses against cyathostomins, thus resulting in the observed low (or absent) parasite burdens. Interestingly, some methanogens (i.e. *Methanosphaera stadtmanae*) have been shown to regulate Th17 responses in mice (Blais Lecours et al., 2011, Bernatchez et al., 2017); in turn, these responses have been linked to the ability of mice to clear experimental infections by *Heligmosomoides polygyrus* (Reynolds et al., 2014b). Mechanistic studies aiming to evaluate the effects of expanding populations of gut methanogens on host mucosal responses and, in turn, GI helminth establishment, may assist the elucidation of these interactions.

An increased abundance of Methanomicrobia in C*low* and C*0* may also be linked to other environmental factors that are simultaneously responsible for the low FEC observed. An example is represented by horse grazing behaviour; indeed, it is known that some individuals within a herd favour less nutritional swards of grass in order to avoid faecal contamination (Hutchings et al., 2000). In turn, as animal faeces often act as fertilisers, individuals favouring nutritious grass are exposed to higher numbers of infective larvae. Grazing different swards of grass may also impact on dietary fibre levels, and thus on gut methanogen populations, as observed in ruminants (McAllister et al., 1996). In horses, dietary factors have also been associated with changes in abundance of Methanomicrobia; for example, *Methanocorpusculum* archaea were observed at a median of 17.7% in horses fed a forage-grain diet, and at a median of 31.9% in horses maintained on pasture (Fernandes et al., 2014). Differences in grazing behaviour between individuals may also be accountable for the increased abundance of bacteria of the Phylum Elusimicrobia in C*0* versus C*200* as these taxa are primarily a component of termite hind-gut microbiota (Gómez and González-Megías, 2007, van Klink et al., 2015, Mikaelyan et al., 2017). Experimental cyathostomin infections of stabled horses may eliminate the effect of grazing behaviour on gut microbial profiles, although ethical concerns may prevent the execution of such studies in the future.

In contrast to uninfected horses, the faecal microbial profiles of C*200* were characterised by an increased abundance of GMD14H09, Phylum Proteobacteria, Class Deltaproteobacteria. Increases in Proteobacteria abundance have repeatedly been reported in association with helminth infections, e.g. in mice infected by *Trichuris muris* and *H. polygyrus*, pigs infected by *Trichuris suis*, and rabbits infected by *Trichostrongylus retortaeformis* (Li et al., 2012, Holm et al., 2015, Cattadori et al., 2016). Proteobacteria are known to increase in the presence of GI inflammation (Shin et al., 2015); hence, the expansion of populations of Proteobacteria in the faecal microbiota of horses with higher infection burdens may be indicative of an inflammatory status of the intestinal tract of these horses at the time of sampling.

One of the objectives of this study was to assess the impact of anthelmintic treatment on the faecal microbial profiling of cyathostomin-infected horses. In particular, ivermectin administration to C*high* was followed by a significant decrease in populations of the Phylum TM7 at D14. Since the relative abundance of TM7 remained unchanged following ivermectin administration in C*low*, it is tempting to speculate that a mutualistic association may exist between TM7 and cyathostomins, whereby each promote establishment of the other, similar to the mutual relationship described for Lactobacillacaeae and *H. polygyrus* (Reynolds et al., 2014a). Bacteria belonging to the Phylum TM7 are obligate epibionts of *Actinomyces* spp. (He et al., 2015), and are thus uncultivable. While TM7 have not previously been linked to GI helminth infections, this phylum of bacteria has been associated with mucosal inflammatory disease in humans (Kuehbacher et al., 2008). Interestingly, TM7 isolates have been shown to repress the induction of TNF-α production in macrophages infected by *Actinomyces odontolyticus*, thus suggesting a potential immune suppressive activity (He et al., 2015); hence, TM7 may promote the establishment of cyathostomins by suppressing effective anti-parasite immune responses. Furthermore, an increase in the taxa *Adlercreutzia* (Phylum Actinobacteria) and R445B (Phylum Lentisphearae) was observed in C*high* at D2 and D14, respectively. The latter was also increased in C*low* at D14, suggesting that this change was unrelated to cyathostomin removal. Bacteria of the genus *Adlercreutzia* produce the metabolite equol (Maruo et al., 2008), a known anti-inflammatory agent and vasodilator (Blay et al., 2010). Thus, it could be hypothesised that increases in populations of *Adlercreutzia* and its metabolites following ivermectin administration might contribute to the emergence of hypobiotic larval stages of cyathostomins (which is known to occur post-anthelmintic treatment; Lyons et al., 2000), via the suppression of effective mucosal immune responses. This hypothesis requires testing in controlled mechanistic experiments.

FEC are often utilised as proxy of parasite infection burdens; however, several investigations have confuted this practice, as weak correlations have been detected between FEC and parasite burdens in horses with >500 e.p.g. of faeces (Nielsen et al., 2010). While the FEC cut-offs used in this study are indicative of differing infection burdens between groups, any inference on the relationships between number of worms in the horse intestine and gut microbial profiling must be taken with caution. Ethical considerations prevent us from performing post-mortem total worm counts in experimentally infected horses; nevertheless, in the future, it may be possible to establish unequivocal relationships between cyathostomin infection burdens (including encysted larvae) and gut microbial profiling from samples collected in an abattoir.

Clearly, a complex network of host-parasite interactions, as well as environmental factors, contribute to the findings reported in this study, and thus further work is needed to disentangle the causality of these relationships. However, one key question that needs addressing is whether differences in host immunity may be associated with significant changes in gut microbial composition (and vice versa) and, if such is the case, whether the horse gut microbiota could be manipulated to improve resistance to helminth infection. Indeed, previous investigations in cattle and mice have reported that host genes encoding for antimicrobial proteins are up-regulated in the mucosae of animals resistant to helminth infection (D'Elia et al., 2009, Li et al., 2015). In addition, dietary supplementation with both pro- (Bautista-Garfias et al., 1999, Bautista-Garfias et al., 2001, Martinez-Gomez et al., 2009, Martinez-Gomez et al., 2011, Oliveira-Sequeira et al., 2014, El Temsahy et al., 2015) and pre-biotics (Petkevicius et al., 2003, Petkevicius et al., 2004, Petkevicius et al., 2007, Thomsen et al., 2005, Jensen et al., 2011), has led to significant reductions in worm burdens in murine and swine helminth infection models, thus indicating that alterations of the gut bacterial flora may bias host immune responses against parasites. Further characterisation of equine host mucosal responses and GI microbiota, in the presence or absence of helminth infection and accompanied by total enumeration of infecting parasites, is a key area of future research, as it may lead to the identification of microbial factors linked to host susceptibility.

In conclusion, cyathostomin infection in horses was associated with global shifts in faecal microbial composition and diversity, in accordance with previous studies in other host-helminth systems, as well as significant changes in specific populations of gut bacteria. Such changes predominantly involved ‘minor’ phyla, thus suggesting that the equine ‘core’ gut microbiota remains unaltered in the presence of burdens of cyathostomins such as those observed in this study. Our findings also support the hypothesis that selected bacterial taxa, and/or their metabolites, may play roles in biasing the host immune response either for, e.g. TM7, or against, e.g. Methanomicrobia, cyathostomin infection. These data pave the way for future mechanistic studies aimed to identify microbial factors linked to host susceptibility, and to manipulate the GI microbiota of horses (e.g. via dietary or probiotic interventions), in order to improve resistance to cyathostomins.
